# Agricultural and Management Practices and Bacterial Contamination in Greenhouse *versus* Open Field Lettuce Production

**DOI:** 10.3390/ijerph120100032

**Published:** 2014-12-23

**Authors:** Kevin Holvoet, Imca Sampers, Marleen Seynnaeve, Liesbeth Jacxsens, Mieke Uyttendaele

**Affiliations:** 1 Laboratory of Food Microbiology and Food Preservation, Department of Food Safety and Food Quality, Faculty of Bioscience Engineering, Ghent University, Coupure links 653, Ghent B-9000, Belgium; E-Mails: kevin.holvoet@ugent.be (K.H.); liesbeth.jacxsens@ugent.be (L.J.); mieke.uyttendaele@ugent.be (M.U.); 2 Laboratory of Food Microbiology and Biotechnology, Department of Industrial Biological Sciences, Faculty of Bioscience Engineering, Ghent University Campus Kortrijk, Graaf Karel de Goedelaan 5, Kortrijk B-8500, Belgium; 3 INAGRO, Provincial Research and Advisory Center for Agriculture and Horticulture, Ieperseweg 87, Rumbeke-Beitem B-8800, Belgium; E-Mail: marleen.seynnaeve@inagro.be

**Keywords:** lettuce, water quality, primary production, pathogens, good agricultural practice

## Abstract

The aim of this study was to gain insight into potential differences in risk factors for microbial contamination in greenhouse *versus* open field lettuce production. Information was collected on sources, testing, and monitoring and if applicable, treatment of irrigation and harvest rinsing water. These data were combined with results of analysis on the levels of *Escherichia coli* as a fecal indicator organism and the presence of enteric bacterial pathogens on both lettuce crops and environmental samples. Enterohemorragic *Escherichia coli* (EHEC) PCR signals (*vt1* or *vt2* positive and *eae* positive), *Campylobacter* spp., and *Salmonella* spp. isolates were more often obtained from irrigation water sampled from open field farms (21/45, 46.7%) *versus* from greenhouse production (9/75, 12.0%). The open field production was shown to be more prone to fecal contamination as the number of lettuce samples and irrigation water with elevated *E. coli* was significantly higher. Farmers comply with generic guidelines on good agricultural practices available at the national level, but monitoring of microbial quality, and if applicable appropriateness of water treatment, or water used for irrigation or at harvest is restricted. These results indicate the need for further elaboration of specific guidelines and control measures for leafy greens with regard to microbial hazards.

## 1. Introduction

Concerns have emerged with regard to the safety of fresh produce in response to some major outbreaks and reported emerging risks linked to fresh produce and derived food products [1,2,3,4]. Disease outbreaks reported in recent years, both in the US and the EU, have particularly been associated with leafy vegetables (spinach, lettuce, and lettuce mixes or salads) [5,6,7,8,9]. *Salmonella* spp. and leafy greens are ranked as the pathogen-food combination identified as the highest concern in the risk-ranking exercise of European Food Safety Authority [10]. Traces back to the farm have confirmed that leafy greens are linked to several outbreaks, although definitive identification of the mode of contamination is largely unknown [11]. In some instances there is evidence of water as the source of microbial contamination. This was the case in the 2006 *Escherichia coli* O157 outbreak linked to bagged spinach in the US [12,13] and in the 2005 *E. coli* O157 outbreak linked to iceberg lettuce in Sweden [7]. In this latter outbreak, the problem strain was isolated from the water source used for irrigation, with the primary source probably being grazing cattle or wild animal activity in the surrounding area.

Overall, pre-harvest contamination of leafy greens can occur directly or indirectly via (wild) animals, insects, water, soil, dirty equipment, and human handling. Other important routes of contamination are the application of manure or compost as fertilizer to fields where crops are grown and fecal contamination of water used for irrigation or pesticide application [14,15,16,17,18,19]. It is assumed that lettuce production in greenhouses is less prone to microbial contamination because greenhouses are protected from the outside environment. The most important contamination sources in the greenhouse are irrigation water and the introduction of manure [20,21]. It is clear that in open fields, the production system is more difficult to control and more prone to contamination as these fields face multiple contamination sources. Risks posed by livestock and wild animals are dependent upon the prevalence, incidence, and amount of pathogen carriage in the animal hosts and the degree of interaction between the animals and the lettuce crop production field [13,22,23,24,25]. Birds are particularly problematic because they have the ability to transmit pathogens over substantial distances and are difficult to control [26,27]. During rainfall or storm events, the topology of the land is crucial as low-lying growing sites are more prone to potential contamination [28]. Climatic conditions and in particular rainfall and temperature are able to impact the release, growth, and survival of fecal indicators along with a variety of pathogenic microorganisms, which may be introduced or maintained for prolonged periods in the production environment [29,30,31].

In a discussion group in 2011, food safety experts from various stakeholder types in the farm-to-fork continuum of the fresh produce supply chain in the EU identified the application of good agricultural practices (GAP) to be the most important control measure to assure the safety of fresh produce [32]. GAPs are defined at the international level in the Codex Alimentarius Commission’s Code of practice for fresh fruits and vegetables (CAC/RCP 53-2003) [33]. To improve the safety and hygiene of primary production, the adherence to GAP is promoted in Europe by EU Regulation 852/2004 and enforced and verified by inspections and audits by national competent authorities [34]. As a valuable instrument to aid individual farmers to implement GAPs, guidelines, manuals, and certification standards were developed at a national or regional level (with or without official approval of competent authorities) and are in use by industry associations, farmer organizations, and retailers. Although these guidelines or standards provide general knowledge and instructions on implementation of GAPs in plant primary production, they are often not tailored to leafy greens or a defined production situation (e.g., greenhouse or open field). Apart from often confidential inspection and audit reports, there is little information or research to identify the status and maturity of the current agricultural practices and management systems in place. Given the overall higher concerns about chemical compared to microbial contaminants by EU consumers [35] and the major attention on integrated pest management and well-elaborated pesticide residue monitoring plans in the fresh produce supply chain, it is not clear to what extent these national or regional guidelines used in Europe on prerequisite programs cover the governance of risk factors for microbial contamination of fresh produce.

The objective of the present study was to get insight on the status of implementation of good agricultural production practices and management systems in place for lettuce production in the region of West Flanders, Belgium. To do this, we used a combination of interview, checklist, and exploitation of microbial data of lettuce crops and environmental sampling (water, soil) from randomly selected lettuce production farms. In addition, as both greenhouse production (almost all year round) and open field production (in the summer period) of lettuce crops is common in that region, we investigated whether the type of production impacted the overall risk factors for microbial contamination.

## 2. Experimental Section

### 2.1. Selection of Lettuce Production Farms

In Belgium in 2009, open field farms producing vegetables covered 39,559 ha of which 12.2% was used for butterhead lettuce (*Lactuca sativa* v. *sativa*). Greenhouse production occupied only 1034 ha of which 22% was used for lettuce [36]. Eight Belgian lettuce production farms active in cultivation of butterhead lettuce were included in this study (Table 1): four greenhouse farms (farms 1 to 4) and four open field farms (farms 5 to 8). Butterhead lettuce is the main lettuce variety grown in Belgium. It is characterized by moderate head weight (400–550 g), soft leaves, and semi-closed head formation. It is commonly marketed in Europe, predominantly as whole heads, but is also available as pre-cut bagged lettuce. The eight farms were all independent family farms located in the region of West Flanders, Belgium. Seven farms were small scale and one farm was a large-scale farm according to the definition of Martins and Tosstorff [37].

**Table 1:** Characteristics of the eight farms in West Flanders, Belgium, used in this study

	Farm 1	Farm 2	Farm 3	Farm 4	Farm 5	Farm 6	Farm 7	Farm 8
Type	Greenhouse	Greenhouse	Greenhouse	Greenhouse	Open field	Open field	Open field	Open field
Size	2.5 ha lettuce	1.75 ha lettuce	0.95 ha lettuce	1.8 ha lettuce	12 ha lettuce	5 ha open field	20 ha ^1^, 2.25 ha ^2^ lettuce	120 ha ^1^, 6 ha ^2^ lettuce
Personnel (approximate)	5	4	3	3	6	2	6	8
Period of production	Whole year	Whole year	September–April	Whole year	April–September	April–October	April–September	May–September
Marketing	Auction	Auction	Auction	Fresh-cut processing Auction	Fresh-cut processing	Auction	Auction	Fresh-cut processing Auction

^1^ Total area for vegetable production; ^2^ area for lettuce production.

### 2.2. Interview on Good Agricultural Practices and Checklist Concerning Water Management

An in-depth interview with the farmers (ca. 3 h) was conducted in 2012 using the self-assessment tool elaborated by Kirezieva *et al.* [38,39] to track the status of implementation of good agricultural practices and the maturity of the management systems in place. The self-assessment tool uses a number of questions related to:

(i) The context of the farm by asking about its product and process characteristics (such as open field *vs.* greenhouses), other activities on the farm (e.g., animal production, applied water sources), and its organization (such as competence and involvement of employees, management commitment, relationship with suppliers and customers);

(ii) The control and assurance activities in place (such as personnel hygiene requirements, control of water supply or water quality, hygienic design of equipment and facilities, application of fertilizers, the use of pesticides, the implementation of a pesticides residue or microbiological monitoring program, the criteria or guidelines used for interpretation of results of analysis, complaints on (visual) quality or safety, availability of procedures, documentation and record keeping, corrective action).

During the interview we noted for each of these aspects whether management and operation of good agricultural practices were absent or present on an unstructured and *ad hoc* basis or a more systematic, formalized, and documented basis; whether it was based on historical self-knowledge, or based upon guidelines or regulatory information, or tailored and validated to the farm’s own situation; if it was supported by any visual checks, sampling and analysis, data collection, and record keeping, and if so whether any trend analysis or remediation or updating occurred on a regular basis. The outcome of this structured interview was applied to gain additional insights in potential risk factors at the farms and combining this outcome with the results of the microbiological survey.

Furthermore, management information in particular with regard to water use and water quality was gathered at the different farms both by observation and completing a checklist. This checklist (Appendix) included questions related to identification, location, and protection of the water source; sampling and testing of microbial water quality; and if applicable, water treatment and its validation.

### 2.3. Microbiological Surveys

Between April 2011 and December 2012, microbiological data were collected on the prevalence of pathogenic bacteria (Enterohemorragische *Escherichia coli* (EHEC)*-vt1* or *vt2* gene and *eae* gene PCR positives, *Salmonella* spp., or thermotolerant *Campylobacter* spp. isolates) and indicator bacteria (total psychrotrophic plate count [TPAC], total coliforms, *E. coli*, enterococci) by sampling three separate lettuce crop production cycles on each farm throughout the production season, as described by Holvoet *et al.* [30]. Coliforms and enterococci were only analysed for water samples. The aerobic TPAC (22 °C) was determined to assess its functionality as an overall utility indicator and correlation to other indicator organisms. The pathogens *Salmonella* spp., EHEC *E. coli*, (*i.e.*, *E. coli* strains possessing the vtx-coding genes *vt1* or *vt2* and the intimin-coding gene *eae*), and *Campylobacter* spp. were analyzed for lettuce crops (and seedlings) as well as for the irrigation water samples. For soil, only *Salmonella* spp. and EHEC were included as pathogens in the analysis. A production cycle is the time required to follow a lettuce crop from the seedling start until its harvest (5 to 14 weeks depending upon the season). Each visit was subdivided into four different sampling moments: at the start of production (planting of the seedlings), approximately 2 weeks and 1 week before harvest, and finally at crop harvest. Sampling included lettuce crops (and seedlings at the start of the crop production cycle) as well as environmental samples, including either peat-soil of the seedlings or field soil surrounding the sampled lettuce crop and irrigation water (taken at the water source and at the tap of the irrigation sprinkler if in use).

Holvoet *et al.* [30] used data from microbial analysis to describe and compile the relationships between levels of hygiene indicator bacteria, detection of enteric zoonotic pathogens, and temperature and precipitation during lettuce primary production. Although in the present study we exploited the same data set, the data set was sorted per type of production situation (greenhouse *vs.* open field production) (Table 2 and Table 3). In addition, the objective of the present study was to compare these two types of production systems and between individual farms. We therefore combined the microbial results with information on the farms’ agricultural (and water) management system as established by the interview and checklist to document and assess this. With this data collection process, a well-founded insight into the status of implementation of good agricultural practices could be achieved.

**Table 2:** Results of greenhouse and open field farms for different microbial indicators isolated from samples in primary production (indicators Total Plate Count [TPAC], *E. coli*, coliforms, *Enterococcus*—in log CFU/g for samples of lettuce, soil, and seedlings or log CFU/100 ml for water samples; pathogens *Salmonella* spp., *Campylobacter* spp., and EHEC—presence or absence/25 g for samples of lettuce, soil, and seedlings or 1 L for water samples) with n = number of samples. Median, minimum and maximum were calculated from the values above detection limit (*i.e.*, *E. coli* ≥ 0.7 log/g or ≥ 0 log/100 mL, coliforms ≥ 0 log/100 mL, enterococci ≥ 0 log/100 mL).

Greenhouse Farms							Open Field Farms				
	n	Prevalence	Med	Min	Max	n	Prevalence	Med	Min	Max
lettuce	TPAC	144	100%	6.3	5.0	8.5	120	100%	6.0	5.0	7.2
	*E. coli*	144	1.4%	0.7	0.7	0.7	120	10%	1.0	0.7	2.0
	Pathogens	48	8.4%				40	10%			
seedling	TPAC	12	100 %	6.2	5.1	6.9	11	100%	5.6	4.6	6.3
	*E. coli*	12	0 %	0.7	0.7	0.7	11	9.1%	1.4	1.4	1.4
seedling soil	TPAC	28	100%	9.0	7.0	9	29	100%	8.0	6.1	9.3
	*E. coli*	28	92.9%	1.7	0.7	3.7	29	100%	2.2	1.4	3.9
	Pathogens	12	0%				11	0%			
soil	TPAC	144	100%	7.2	6.3	8.3	132	100%	7.1	6.0	8.9
	*E. coli*	144	38.2%	1.2	0.7	2.9	132	34.8%	1.2	0.7	3.2
	Pathogens	48	4.2%				44	9%			
water source	TPAC	35	100%	5.0	2.7	7.2	33	100%	5.9	4.8	7.1
	*E. coli*	35	48.6%	1.0	0	1.9	33	0%	2	1.0	3.6
	Coliforms	35	31.4%	1.0	0	3.5	33	0%	2.3	1.0	4.1
	*Enterococcus*	35	45.8%	1.3	0	2.5	33	0%	1.9	0.6	3.6
	Pathogens	35	20%				33	54%			
water tap	TPAC	36	100%	5.3	2.3	7.8	5	100%	6.7	5.8	7.7
	*E. coli*	36	19.4%	1.1	0	1.7	5	0%	2	1.5	2.1
	Coliforms	36	27.7%	0.7	0	2.1	5	0%	2.1	1.5	2.7
	*Enterococcus*	36	33.3%	0.9	0	2.3	5	0%	2.0	1.7	2.7
	Pathogens	36	2.8%				5	20%			
wash water	TPAC	4	100%	5.5	4.3	6.4	7	100%	6.3	5.7	7.7
	*E. coli*	4	75%	0	0	0.3	7	71.4%	0.9	0.8	1.5
	Coliforms	4	75%	0.1	0	0.3	7	71.4%	1.2	0.9	1.45
	*Enterococcus*	4	50%	0.2	0	0.5	7	71.4%	0.6	0.3	1.0
	Pathogens	4	0%				7	57%			

**Table 3:** Pathogen prevalence in lettuce, soil, and seedling samples (presence /25 g) and in water samples (presence per liter) taken in greenhouses and open field farms.

		n ^a^	Pathogen		PCR Screening ^b^	Confirmed by Culture
GREENHOUSE FARM	Lettuce	48	*Campylobacter* spp.	8.4%		
	Soil	48	EHEC	4.2%	*vt1*, *eae*	
	Soil	48	EHEC	4.2%	*vt2*, *eae*	
	Water source	35	*Salmonella* spp.	2.9%		
	Water source	35	EHEC	2.9%	*vt1*, *vt2*, *eae*	
	Water source	35	*Campylobacter* spp.	20%		
	Water tap	36	*Campylobacter* spp.	2.8%		
OPEN FIELD FARM	Lettuce	40	*Campylobacter* spp.	10%		
	Soil	44	*Salmonella* spp.	2.4%		
	Soil	44	EHEC	6.8%	*vt2*, *eae*	O157
	Soil	44	EHEC	6.8%	*vt1*, *vt2*, *eae*	O103, O157
	Soil	44	EHEC	6.8%	*vt1*, *eae*	O26
	Water source	33	EHEC	15.2%	*vt1*, *eae*	
	Water source	33	EHEC	15.2%	*vt1*, *eae*	O111
	Water source	33	EHEC	15.2%	*vt1*, *eae*	O26
	Water source	33	EHEC	15.2%	*vt1*, *vt2*, *eae*	
	Water source	33	EHEC	15.2%	*vt1*, *eae*	
	Water tap	5	*Campylobacter* spp.	20%	**	
	Wash water	7	*Campylobacter* spp.	57.1%		

^a^ Number of samples checked; ^b^ PCR screening with Genedisc or the method of Posse *et al.* [41]; EHEC, *E. coli* strains possessing the vtx-coding genes *vt1* or *vt2* and the intimin-coding gene *eae.*

### 2.4. Data Processing and Statistical Methods

Results were compiled and graphs were made in Excel. Many of the *E. coli* enumerations for lettuce, soil, or water were expected to be negative, *i.e.*, values below the detection limit. For statistical analysis, the *E. coli* data set was transferred into classes defined as follows: class 1, <0.7 log CFU/g or 0 log CFU/100 mL (undetected); class 2, ≥0.7 and <2 log CFU/g or ≥0 and <1 log CFU/100 mL; class 3, ≥2 and <3 log CFU/g or ≥1 and <2 log CFU/100 mL; and class 4, ≥3 log CFU/g or ≥2 log CFU/100 mL. IBM SPSS Statistics 20 and Microsoft Excel were used for statistical analysis. For the mean, minimum, and maximum calculations, only samples with numbers higher than the detection limit were included in the analysis (≥0.7 log CFU/g or ≥0 log CFU/100 mL). For the comparison of *E. coli* prevalence between greenhouses and open field farms and between sample type, the Pearson chi square (PC) or Fisher’s exact test (FET) were used in case one group contained less than five samples (*P* < 0.05).

The Kolmogorov-Smirnov test and Levene’s test were used to assess normality and equality of variance (*P* ≥ 0.05), respectively. If normality could not be assumed, the Mann–Whitney U test (MW) was used; in the case of normality, a *t*-test was used. To determine the relation between the notation of water treatment from the checklist and microbial contamination of the water as determined by analysis, the Wilcoxon signed-rank test was used.

## 3. Results

### 3.1. Context, Organization & Management Practices of Lettuce Production Farms

**Figure 1:**
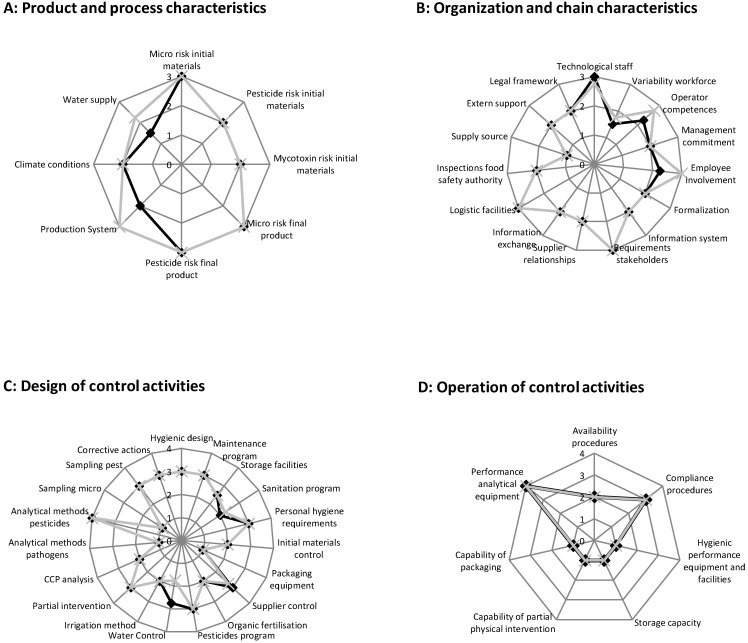

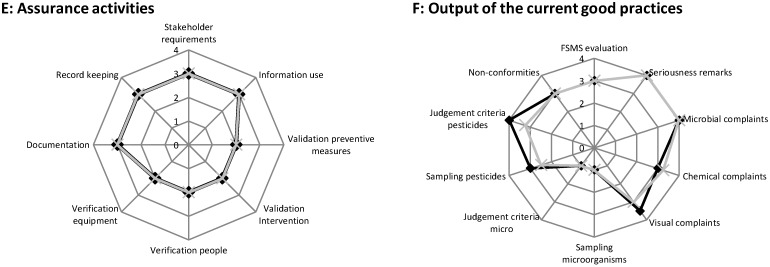
Spiderwebs demonstrating the mean of the results of the self-assessment tool on current agricultural practices and management systems of the eight farms, greenhouse farms ■ and open field farms **×** **A**, **B**: Level 1 low-risk, level 2 medium-risk, and level 3 high-risk situations. **C**, **D**, **E**: Level 1 activity is not present; level 2 activity is conducted based on historical knowledge of the farmers based on self-insights; level 3 activity is performed based on best practices according to guidelines; and level 4 activity is tailored and fit-for-purpose for the farm-specific situation. **F**: Level 1, no information is available; level 2, ad hoc information is present and sometimes problems occur (reactive behavior of farmers); level 3, systematic information is collected and sometimes problems occur (proactive behavior of farmer); level 4, systematic information is present and no problems are occurring.

The results of the self-assessment tool are displayed in spiderwebs in Figure 1A–F as calculated means per indicator for the greenhouses (n = 4) and open field farms (n = 4). All farms in the present study were independent family farms that deliver the lettuce immediately after harvest (same day within 6 h or exceptionally by the next day) in plastic crates (usually 12 crops per crate) to the auction (within 30 km). Two companies also sold directly to nearby fresh-cut processing companies (Table 1). In most cases the lettuce was stored at the farm under controlled refrigerated conditions, but transport occurred by truck to the auctions or fresh-cut companies under uncontrolled ambient conditions. Therefore, the product and process characteristics shown in Figure 1A are similar for all farms.

Seven farms were small-scale farms and one open field farmer was a large producer. The workforce was between two and eight workers (including the farm owner and his wife) (Table 1). Three out of four open field farms (>2 ha) had a high personnel turnover and used foreign seasonal workers with more difficulties of employee involvement and hygiene training, whereas one open field farmer (< 2 ha) and the four greenhouse farmers used a stable work force with native workers. This was reflected in the questions on “employee involvement” and “operators competences” (Figure 1B).

The management system in elaborating good agricultural practices based on control and assurance activities (Figure 1C–E) was mainly based on the Belgian national sector guidelines and recommendations laid down in the IKKB standard [40] and was approved and recognized by the Belgian food safety agency to encompass all minimum legislative requirements (e.g., application of manure, selection source of water, personnel hygiene during manual handling of commodities, requirements for toilets at the farm, and pest control at the farm). That is why the majority of the questions on design of control activities (Figure 1C), actual operation of these control activities (Figure 1D), and assurance activities (Figure 1E) are conducted on level 3.

With respect to the output of the current practices, as illustrated in Figure 1F, all farms were audited on a yearly basis by third parties, and no serious remarks were given as they complied to the current requirements in European and national legislation and IKKB standard. Certification to IKKB standard is a prerequisite in order to be able to deliver lettuce crops to the auctions and further sales to major retail shops or fresh-cut lettuce processing companies. There were, in some cases, extra (sometimes conflicting) requirements set by various retailers in particular with regard to the demand to provide lettuce with maximum pesticide residues lower than the legal Maximum Residue Levels (MRLs) for pesticide residues (Figure 1B, question on “requirements stakeholders” on level 4). There is a high focus on IKKB standard and awareness from individual farmers on appropriate use of pesticides and full registration and documentation of their use (Figure 1C,F). In addition, farmers responded to being aware of extensive efforts by the auctions and the competent authorities to carry out a comprehensive sampling plan for monitoring and providing regular feedback on the pesticide residue testing.

### 3.2. Agricultural Practices (Control and Assurance Activities)

Fertilizer application and irrigation water are known as risk factors for bacterial contamination on leafy greens. Therefore, the results of the self-assessment questionnaire and the water questionnaire (Appendix) on these topics are discussed further.

Two greenhouse farms and one open field farm made use of commercial organic dry pellets and inorganic synthetic fertilizer, both provided by wholesalers. Another open field farm used composted cow manure from the stable to fertilize the field. From the interview with this latter farmer, it seemed that no particular attention was paid to waiting times, although fertilizer applications always occurred at least 2 weeks before planting the seedlings and no fertilizers were applied during the crop cycle. When inorganic fertilizer is used, it is easier to control the release of nutrients compared to the pellets or organic fertilizer for which nutrient dissolution can vary depending upon (wet) weather conditions making it less predictable.

**Figure 2:**
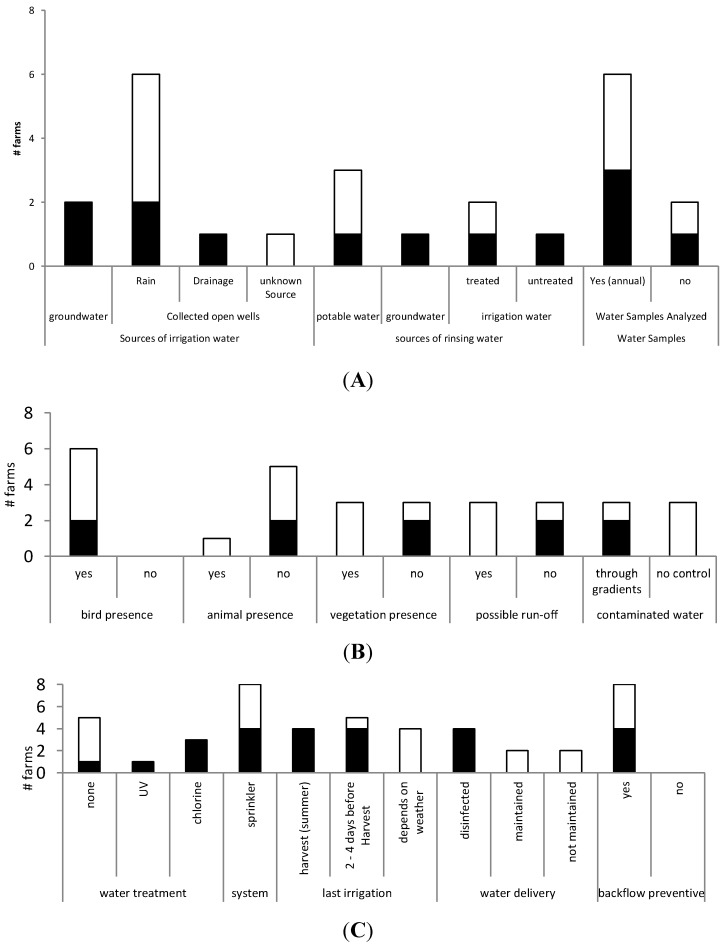
Results of the water management questionnaire (Appendix); black boxes, greenhouses; white boxes, open field farms. **A**: Water sources applied for irrigation, sources of rinsing water, and microbial analysis of the applied water. **B**: Measures taken to prevent contamination routes; farms 3 and 4 were omitted because borehole water was used. **C**: Irrigation method/applied water treatment; *y*-axis, number of farms.

In general, greenhouse farms applied more effort to control water supply and quality compared to the open field farms because of the control of phytopathogens able to induce disease in the lettuce plants (Figure 1A). The greenhouse farms used borehole water (n = 2) or collected rainfall water (n = 2), while all open field farms applied collected rainfall water (n = 4). The open reservoir of one open field farmer was additionally supplied by water of an unknown source (Figure 1C and Figure 2).

The two greenhouse farms using rainwater collected in a reservoir had reservoirs constructed with elevated ditches to prevent run-off water to intrude. In contrast, only one out of four open field farms had an elevated reservoir; thus for the other three there was a potential risk of run-off water in the water reservoir. Also, three out of four greenhouse farms (including one of the greenhouse farms using borehole water) used a water treatment system throughout the whole growing season (Figure 2). Two farmers used chlorine, while the other farmer used ultraviolet (UV) disinfection.

Six farms performed an annual microbial analysis of their water quality to comply with the demand of an annual test result on “clean” water as defined by IKKB guidelines. This is needed if the water is used for rinsing the harvested lettuce heads. Two farms (one greenhouse and one open field farm) did not have any records on water quality as one used borehole water, which was assumed to be of potable water quality, and the other used municipal tap water. With regard to the time interval between the last irrigation event and harvest, greenhouse farms were still being irrigated on the same day of harvest during summer and 2 to 4 days before harvest in winter. For all open field farms, this time interval largely depended upon weather conditions (Figure 2; the summer of 2012 when interviews and sampling was performed was characterized by regular precipitation events) (Figure 1C).

### 3.3. Microbiological Data on Lettuce Production: Greenhouse versus Open Field Farms

From April 2011 to December 2012, 844 samples were collected at eight farms (per three crop production cycles per farm [per three]): 57 peat-soil seedling samples, 23 seedling leaf samples, 264 lettuce head samples (= 792 samples pooled per three), 276 soil samples (= 828 samples pooled per three), 120 water samples, 48 workers’ hands, and 56 transport boxes [30]. The overview of results for the greenhouse farms *versus* the open field farms are shown in Table 2 and Table 3.

**Figure 3:**
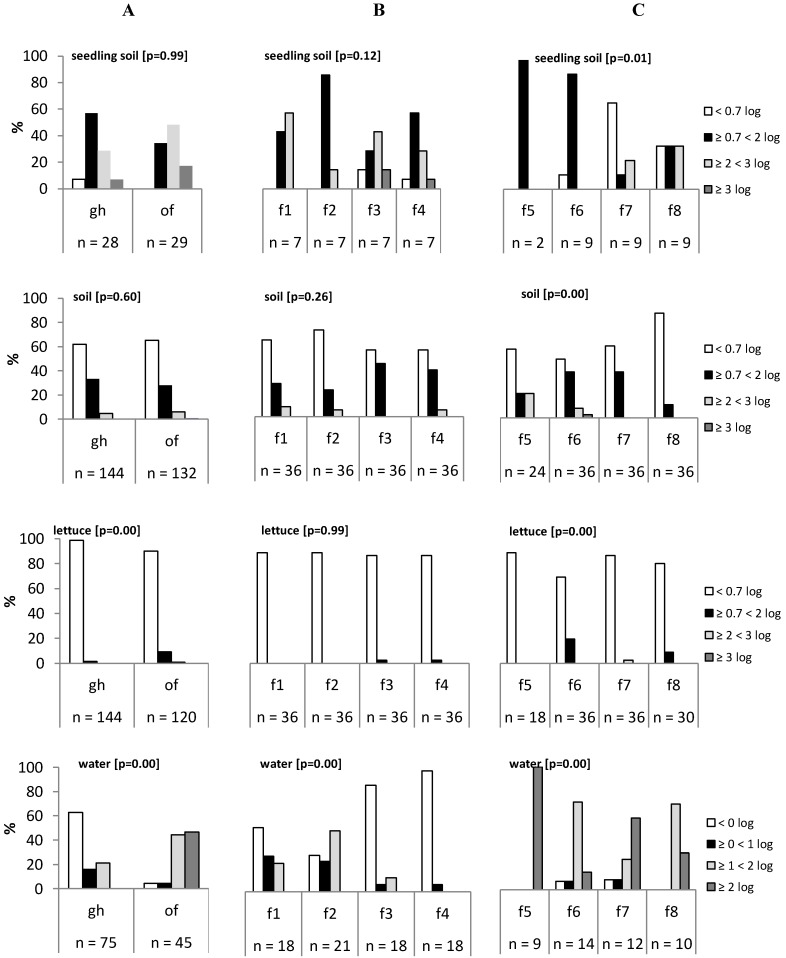
(**A**) Comparison of *E. coli* distribution between greenhouse farms (gh) and open field farms (of) for seedling soil, mature plant soil, lettuce, and water. (**B**) Comparison of *E. coli* distribution between individual greenhouse farms (f1, f2, f3, f4). (**C**) Comparison of *E. coli* distribution between individual open field farms (f5, f6, f7, f8). The P-value is shown after the designation.

For the peat-soil of the seedlings and the samples of the field soil, no difference in *E. coli* class was found between the greenhouse and the open field farms (*P* > 0.05, FET) (Figure 3A). In contrast, the *E. coli* load of the lettuce and the water was significantly different between the two production systems (*P* < 0.05, FET); in approximately 99% of the greenhouse lettuce samples, no *E. coli* was enumerated (< 10 cfu/g) in contrast to 90% of the open field farms (Table 2, Figure 3A). In 39.2% of the greenhouse water samples, *E. coli* was below the detection limit (< 1 cfu/100 mL), while 91.1% and 46.7% of the water samples of the open field farms were higher than 1 log CFU/100 ml and 2 log CFU/100 mL, respectively. The TPAC of the lettuce was significantly higher for the greenhouse farms (median 6.3 log CFU/g) compared to the open field farms (median 6.0 log CFU/g) (*P* < 0.05, *t*-test) (Table 2). Nevertheless, the microbiological relevance of a 0.3 log difference might be limited in terms of microbial quality.

The pathogens (thermotolerant *Campylobacter* spp. and *Salmonella* isolates or EHEC PCR signals) were significantly more frequent in water samples of open field farms (46.7%) compared to greenhouse farms (12.0%) (*P* < 0.05, PC). In other types of samples (soil or lettuce), the pathogens’ prevalence was higher in open field samples compared to greenhouse farms, but this was not significant. On lettuce leaves, *Campylobacter* was the single pathogen detected (n = 4 out of 40 for greenhouse samples and n = 4 out of 48 for open field samples); no EHEC PCR signals or *Salmonella* isolates were obtained (Table 3).

Among the greenhouse farms, no statistical significant difference was observed for the *E. coli* classes (all *P* > 0.05, FET) of seedling soil, mature plant soil, and lettuce samples; the same distribution was observed for all greenhouses (Figure 3B). The results of microbial analyses of farms 3 and 4 had a different distribution in contrast to the other two farms, probably attributable to the difference in the type of water source being used (groundwater water *versus* open well water); this difference was significant (*P* < 0.05, FET).

A significant difference in *E. coli* class was found for all types of samples among the four open field farms (*P* < 0.05, FET) (Figure 3C). For example, the lettuce samples of farm 5 were all below detection limit, while *E. coli* was enumerated in approximately 20% and 10% of the samples from farms 6 and 8, respectively. A higher variability in *E. coli* levels among the open field farms was found in seedling soil, mature plant soil, lettuce, and water.

### 3.4. Impact of Agricultural Practices and Management Systems on Microbial Quality

There was no difference in the number of *E. coli* between the soil samples at the start of the production between the farms that used commercially available organic pellets (farms 2, 4 and 7) and the farms that used inorganic fertilizer (farms 1, 3, 6, and 8) (*P* > 0.05, FET). An increased number of soil samples with elevated levels of *E. coli* were observed for farm 5, which used cow manure, compared to the farms that used organic pellets and inorganic fertilizer (*P* < 0.05, FET). However, no difference was observed for the soil samples among the farms that used organic dry pellets, inorganic fertilizer, or cow manure when the samples were taken later in the crop production cycle (*P* > 0.05).

**Figure 4:**
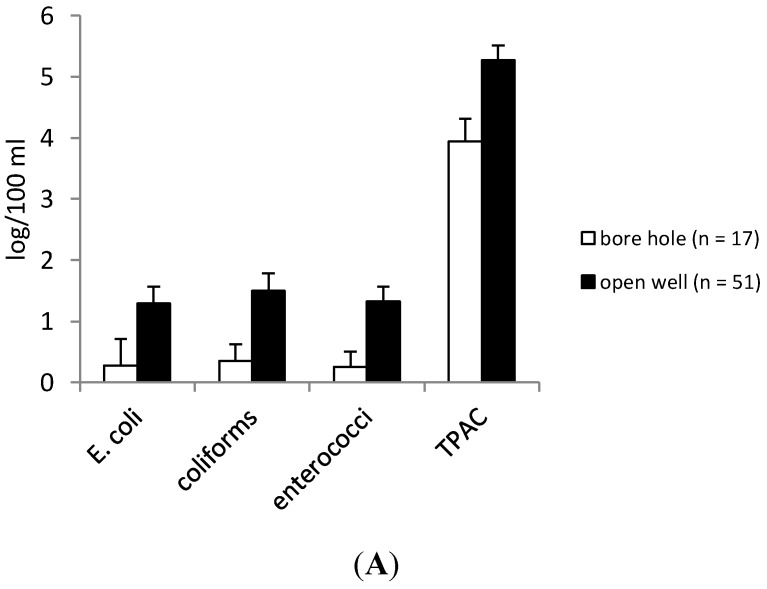

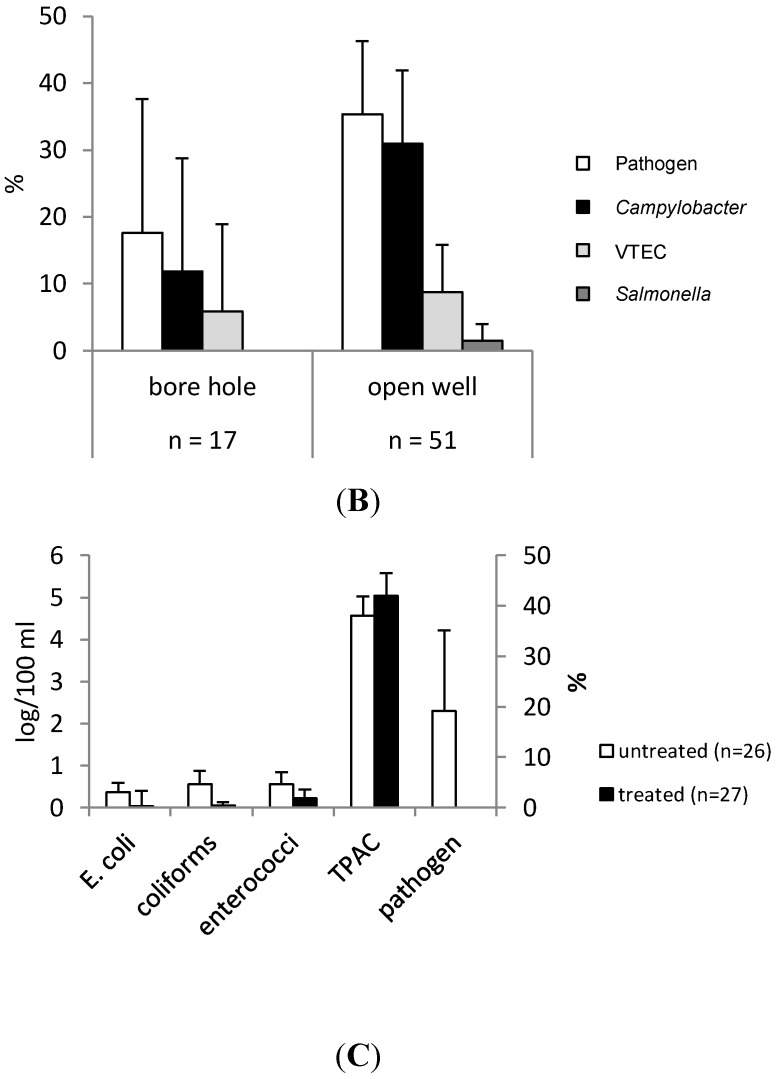
Degree of contamination of the different indicator bacteria (log CFU/100 ml) (**A**) and pathogens (presence/absence in 1 l) (**B**) for borehole water (farms 3 and 4) and open well water (farms 1, 2, 5–8). (**C**) Impact of water treatment on the indicator bacteria and pathogens by comparing treated and untreated water for three farms applying water treatment (farms 1, 3 and 4). Bars show the 95% confident interval.

Of the eight farms, two farms used borehole water as the water source for irrigation compared to open well water for the other six farms. There was a significantly higher number of samples with elevated levels of *E. coli*, coliforms, enterococci, and TPAC in the open well water compared to the borehole water (*P* < 0.05, MW and *t*-test for TPAC) (Figure 4A). The prevalence of pathogens was also lower in the borehole water compared to the open well water (Figure 4B).

Three out of four greenhouse farms used some water disinfection method between the source and tap (Figure 2). No pathogens were observed in the water sampled at the tap in contrast to the water sampled at the source (in the water reservoir). Overall, lower numbers of *E. coli* and enterococci were observed in the water samples taken at the tap, whereas overall higher numbers of TPAC were obtained in the tap water samples when compared to TPAC numbers of the water sampled at the source (Figure 4C).

## 4. Discussion

Overall, EU consumers have expressed more concern about chemical than microbial contaminants [32,35]. Therefore, the focus for fresh produce (including leafy greens) is on chemical hazards during primary production, processing, and trade in Europe. In 2005, Regulation (EC) No. 396/2005 became effective; this promoted a harmonization of the MRLs for pesticides at the EU level [42]. EU Member States are obliged to ensure compliance with EU MRLs and have extensive monitoring programs in place for fruit and vegetables to check for compliance with the maximum pesticide residue levels in fresh produce.

However, leafy greens are also prone to microbial contamination as demonstrated by multiple outbreak reports, mostly in the US and to a lesser extent in the EU. An example is the 2011 EHEC outbreak in Germany/France that was epidemiologically linked to sprouted seeds [43]. This outbreak raised media attention and concerns among EU consumers [44]. Still, in interviews performed on the farms during the present study (one year after the 2011 EHEC crisis), limited awareness or knowledge was apparent for human enteric pathogens, such as EHEC, *Salmonella* spp., or *Campylobacter* spp., as potential hazards associated with lettuce, although the January 2011 discussion forum with European stakeholders in the fresh produce supply chain (taking place before the event of the EHEC 2011 crisis) mentioned bacteria as the first threat, followed by viruses and pesticides [32]. Despite the increased awareness of microbial safety of fresh produce among consumers, retailers, the fresh-cut processing industry, farmers’ associations, and competent authorities, there is no EU-wide harmonized microbial monitoring program in place. More profound data are available from published surveys, such as the survey in the Netherlands from 2006 to 2007 on a variety of fresh produce and ready-to-eat salads [45] that demonstrated 0.38% of samples (n = 1860) carried *Salmonella*. Overall, individual national or regional surveys differ in both focus and sampling design, making data comparison at the level of specific food-pathogen combinations inappropriate [10]. In Belgium, collective monitoring plans are in place, e.g., by national competent authorities and the auctions.

Furthermore, at the EU level the current microbiological criteria in place (a process criterion for *E. coli* and a food safety criterion for *Salmonella*, described in EU 2073/2005 Regulation [46]) are only applicable for pre-cut ready-to-eat vegetables and not applicable at harvest for primary production or whole crops being marketed, as sampled in the present study. As a result and confirmed by the results from the self-assessment tool in the system output questions (Figure 1F), individual farmers rarely get complaints or questions about hygiene or microbial safety of lettuce. If complaints were expressed to the farmers, these related to visual quality with slightly more complaints being expressed to open field farms compared to the greenhouse farms.

Although the prevalence of pathogens, such as *Salmonella* and EHEC, are overall very low (< 1%) [45,47], *Salmonella* spp. was still identified as of high concern for being associated with leafy green outbreaks in the EU [47]. In the present study, no *Salmonella* was isolated from greenhouse lettuce or open field lettuce, although *Salmonella* was isolated once from soil in the open field and once in the water source (open well water) from a greenhouse. In addition, the present study showed a higher presence of thermotolerant *Campylobacter* in lettuce and water samples. The combined results of the interviews, checklist, and microbiological analysis indicate the need for further elaboration of specific guidelines and control measures for leafy greens with regard to microbial hazards. For example, the water management checklist showed little knowledge of microbial quality of water used for irrigation or rinsing at harvest and a lack of guidelines on this for the farmers.

Greenhouses and open field farms differed in their production environment. Greenhouses provide physical barriers against some sources of enteric bacterial contamination and more often use water treatment, which may explain the lower variability in microbial results among greenhouse farms and overall lower numbers of *E. coli* compared to open field farms. The open field farms might face additional routes of contamination, such as the introduction of enteric bacteria via neighboring livestock, wild animals, heavy rainfall, or storm events causing run-off or flooding [24,31,39,48].

The most probable origin of micro-organisms of fecal origin in the greenhouses was identified to be irrigation water as well as the introduction of potting soil or dry organic pellet fertilizer despite the fact that the latter potting soil and fertilizer were commercially obtained and treated and would not be expected to contain *E. coli*. Both greenhouses and open field farms suffered from high levels of fecal contamination (up to 3.9 log CFU *E. coli*/g) from incoming potting soil. Few reports document microbial contamination of soil used for raising seedlings. *Salmonella* has been found in supposedly sterilized animal byproducts used in potting mixes [49], and *Legionella* spp. have also been recovered from potting soil [50,51]. The presence of high levels of *E. coli* (up to 2 log CFU/g) in the (initial) soil of the greenhouse farms could be explained by the highly contaminated potting soil as the soil is mixed thoroughly after harvesting and in some cases the next day, the new seedlings were already planted. This has been the case to a lesser extent for the open field farms because the time between harvest and start of the next crop cycle is minimally 2 to 3 weeks and the bacteria experience more stress and competition compared to greenhouses due to the higher humidity and soil moisture, which favors the survival of bacteria [52,53,54].

**Figure 5:**
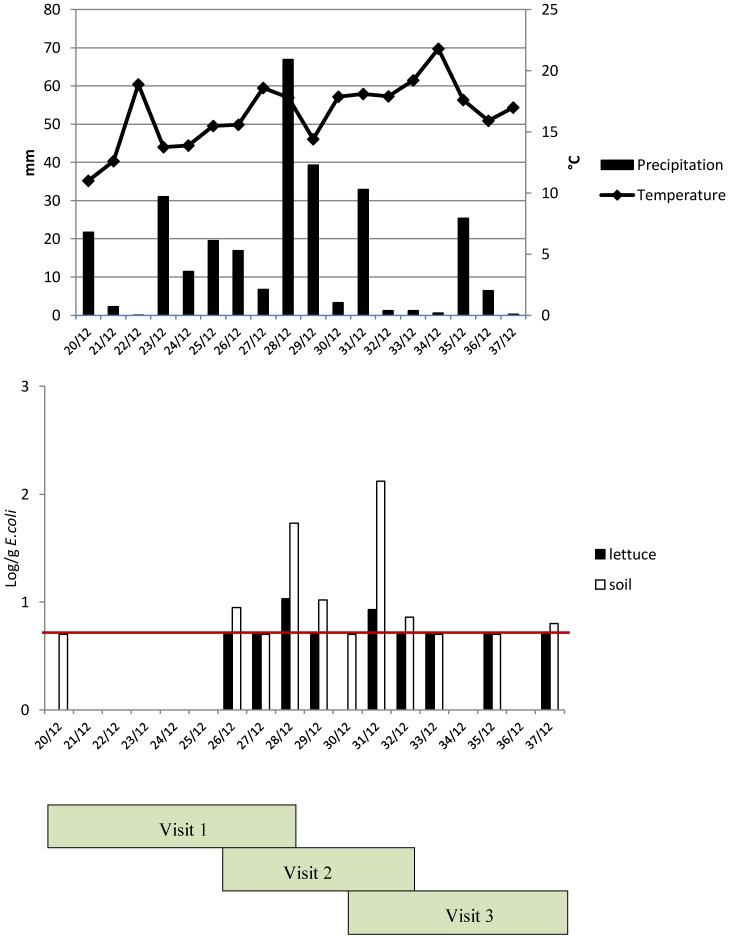
Impact of precipitation and temperature on the presence of indicator bacteria in soil and lettuce during the three visits (starting in week 20 with visit 1 up to week 37 with visit 3) of farm 6; the red line is the detection limit (0.7 log/g).

The lack of any difference in the *E. coli* presence in the soil between farms that used organic fertilizer and those that used inorganic manure suggest that properly handled and treated organic fertilizer, *i.e.*, commercially available dry pellets in the current study, is effective and safe [55]. However, the higher *E. coli* content of the initial soil of farm 5 and the presence of three culture-confirmed PCR EHEC signals on this farm are probably due to untreated or improperly treated farmyard cattle manure [55]. Untreated or improperly treated manure may harbor pathogenic bacteria, such as *Salmonella* spp., *E. coli* O157 H7, *Campylobacter jejuni*, *Yersinia enterocolitica*, and *Clostridium perfringens*, and can contaminate the soil [55,56]. Still, it was among the farms using inorganic fertilizer that the single positive soil sample of *Salmonella* spp. was found (farm 8). In contrast to greenhouse farms, the soil of an open field farm was stated to be more susceptible to contamination from the outside [39]. For example, the low-lying field of farm 6 was flooded during heavy rainfall, and this probably explains the peaks of *E. coli* in the soil (up to 3.5 log/g) and lettuce (up to 1.5 log/g) during sampling moments after heavy rainfall (Figure 5A,B).

In general, there was a higher risk in the water supply for the open field farms compared to the greenhouse farms (Figure 2). The water source of farms 3 and 4 and of the other six farms was different; very low levels of fecal contamination and pathogens were detected in the borehole water during the current study for these two farms. Several studies confirmed our findings that borehole water can be contaminated with different kinds of micro-organisms, such as *E. coli, Salmonella* spp., and *Campylobacter* spp. [57,58,59]. However, borehole water is generally considered to be of better quality because the water is more separated from contamination than surface water [59]. The other six farms used the cheaper alternative—rainwater collected in an (foiled) open well (surface water). Rainfall water is freely available and harvesting may serve as an alternative solution due to the pressure on the borehole water when properly stored [60].

The water control differed between the greenhouses and open field farms (Figure 2). Water samples from farms that used protective walls to avoid contamination from run-off had a lower microbial load. The historic presence of cattle near the open well of farm 5 resulted in a high prevalence (50%) of PCR EHEC signals in the open well water samples. The water reservoir of farm 7 contained rainfall water and water from another unknown water source that was flowing into the open well because the open well was not elevated or protected from intrusion by an external water source. The lack of control of the water was reflected by the high prevalence of pathogens detected in the water (66%). The other farm (farm 6), which used no gradients or protection, also contained high numbers of pathogens in the water sampled either at the source or at the taps for irrigation; in addition, rinsing water used at harvest showed a high prevalence of *Campylobacter* spp. (64%).

Furthermore, three out of four greenhouse farms always used a water treatment system, whereas one farm (farm 2) only provided water treatment in winter (Figure 2). Water treatment can be applied when a more contaminated source is used or to tackle contamination from biofilm formation in the irrigation pipes [61,62]. However, interviewees mentioned that the purpose of the water treatment was mainly to eliminate phytopathogens, such as *Pseudomonas cichorii,* which is known to cause bacterial midrib rot [63,64]. Information from former studies in Belgium indicate that *P. cichorii* is most likely introduced into a greenhouse via contaminated irrigation water [65]. Although the main idea for water treatment is not the elimination of human pathogens, the water treatment applied was able to significantly reduce *E. coli*, enterococci, and coliform levels in the water (*P* < 0.05, Wilcoxon). The occurrence of pathogens was also reduced, which could be expected from UV or chlorine as a treatment [59,66]. In contrast, an increase in TPAC was observed between the water at the source and the water at the tap (at the actual point of irrigation). This could be attributed to biofilm formation in the pipelines since disinfection or maintenance is only annually performed at most farms [67,68].

All farms in this study used sprinkler irrigation (Figure 2). Subsurface or drip irrigation lowers the risk of transfer to growing plants by minimizing the exposure of the irrigated water to the crop compared to sprinkler irrigation [69,70,71,72]. However, the (investment) cost for a drip irrigation system is significantly higher than that of sprinkler irrigation [73]. On every occasion that *Campylobacter* spp. was detected on the lettuce, the *Campylobacter* pathogen was also isolated from the corresponding applied irrigation water, which suggests irrigation water as a route of contamination of lettuce. These findings are supported by several studies confirming that water used for irrigation can transfer human pathogens to a variety of growing leafy vegetables and herbs [11,72,74,75,76,77] and may cause outbreaks [7,78].

At harvest, lettuce was primarily rinsed to remove soil and to reduce to some extent microbial load [79,80]. Water is a useful tool for reducing potential microbial contamination, but rinsing water of insufficient quality has the potential to be a direct source of contamination and a vehicle for spreading microbial contamination [81,82,83]. Although three farmers claimed to use potable water quality for rinsing (Figure 2), only farm 4 satisfied potable water quality [84]. The rinsing water of farms 6 and 7 tested positive for *Campylobacter* spp. because the water was applied at harvest to the whole head, which was then directly transported to the auction or processing company. It is a prerequisite to use clean and preferably potable water, as mentioned in European legislation [34], and *E. coli* or pathogen contamination should then not occur.

Greenhouse farms irrigated in the summer months at the day of harvest (Figure 2) had an increased risk of pathogen presence at harvest in particular because microbial water quality of irrigation water is unknown (and at present also not subject to legislation or microbial guidelines). It is recommended to notably increase the interval from the time of irrigation to the point of harvest due to the decreased likelihood that the pathogen would be present in the harvested product [85,86]. Ottoson *et al.* [87] performed a quantitative microbial risk assessment in Sweden and found that waiting times of 1, 2, 4, and 7 days reduced the risk for *E. coli* O157 contamination by 3, 8, 8, and 18 times, respectively.

Although no difference in contamination was found for the field soil and potting soil samples between greenhouse and open field farms, *E. coli* contamination of lettuce in greenhouses was lower but a higher TPAC level was observed. The lettuce of open field farms was exposed to higher UV radiation, which probably lowers the microbial load and is the most plausible reason for the significantly lower TPAC levels. Under clear skies, UV light can effectively kill microbes [88]. The amount of UV radiation at the surface results from ozone concentrations in the upper troposphere and lower stratosphere, cloud cover, and aerosol type, content, and distribution [89].

On the other hand the flooding and lack of stability and control of the watering process had a negative impact on the contamination level of the open field farms. Increased rainfall or irrigation enhances the chance of flooding or splashing of (contaminated) soil on vegetables compared to regular vaporization of irrigation water used in greenhouses [90,91,92,93,94].

However, in this study there was less need of irrigation in the open fields due to the sufficient amount of precipitation in the summer of 2012 (Figure 5A). An additional reason for the higher fecal contamination of soil, lettuce, and water for the open field farms may have been the time of sampling. All open field farms were sampled during the warmer summer months (May to September), whereas the greenhouses were sampled throughout the year. The prevalence of pathogens was observed to be overall higher in periods of increased temperature and probably also during increased wildlife activity [30].

## 5. Conclusions

The combination of a self-assessment interview on good agricultural practices and management systems in place, water management checklist, and microbiological data enabled us to obtain insight in the quality and safety of lettuce and the agricultural and management practices of lettuce production in the region of West Flanders, Belgium. Although there was knowledge and control of phytosanitary aspects and plant pathogens by the farmers, awareness and knowledge on human pathogenic microbiological hazards was limited. There is a need for further improved national guidelines and creating farmer awareness with more focus on the risk of human enteric pathogens. This would result in better guidance and communication on source, quality, testing frequency, treatment and use of irrigation water and methods of irrigation and on the construction and maintenance of irrigation water reservoirs. The open field farms showed a higher prevalence of pathogens and overall more samples with elevated levels of *E. coli* compared to the greenhouse farms, probably because of the additional external contamination sources. However, in general, greenhouse farms did more to avoid microbiological contamination. Their measures for control of irrigation water quality and protection of reservoirs from external contamination were more advanced due to the application of water treatment and precautions, such as the use of elevated ditches to avoid introduction of run-off water.

Knowing that 45% of the water source samples from the farms without water treatment contained a pathogen, the absence of a water treatment system can have detrimental consequences, in particular for lettuce production in open fields when more irrigation is necessary during dry sunny weather (the present survey in 2012 needed limited irrigation because of regular precipitation). The importance of water quality for the rinse step at harvest is also a critical point; however, it was noted that most farmers did not use potable water or had no guarantees on the cleanliness of the water used. It could be this rinsing step that poses a direct risk for the at-harvest introduction of enteric pathogenic bacteria and thus may impact microbiological quality and safety of the lettuce for the fresh market or fresh-cut processing companies.

## Appendix: Water Management Questionnaire

### Part 1: Water Sources

1. What is/are the sources of irrigation water for this farm? You can give multiple answers

○ Borehole water - closed wells
○ Surface water
  ○ Canal
  ○ Creek
  ○ River
○ Collected open well
  ○ River transfer
  ○ Rain
  ○ Waste water
○ Municipal waste water
○ Industrial waste water
○ Others
  ○ Drainage water
  ○ …

2. Are water samples analyzed for each water source for monitoring the microbial water quality?

○ Yes
  ○ Annual
  ○ Semestrial
  ○ Monthly
  ○ Weekly
○ No

3. What is/are the sources of rinsing water for this farm?

○ Potable water
○ Borehole water
○ Irrigation water
  ○ treated
  ○ untreated
  ○ River
○ Other: …

### Part 2: Preventive Measurements

4. Is there a possibility for presence of birds or bird feces around the water source?

○ Yes
○ No

5. Is there a possibility for presence of other animals and debris around the water source?

○ Yes
○ No

6. Is surrounding vegetation present around the water source?

○ Yes
○ No

7. Is there a possibility for run-off water in the water source?

○ Yes
○ No
  ○ Through lining of canals and well heads
  ○ Redirection of contaminated water with diversion dikes, gradients, inlet/outlet control structures
  ○ Other actions: …

### Part 3: Irrigation Method/Water Treatment System

8. Which water treatment do you apply between the water source and the irrigation system?

○ None
○ Water filtration
○ Chemical sanitizers
  ○ Chlorine
  ○ H_2_O_2_
  ○ Others: …
○ Coagulation + flocculation
○ UV
○ Others: …

9. Which irrigation method do you apply?

○ Furrow/flood irrigation
○ Sprinkler/spray irrigation
○ Drip irrigation
○ Manual irrigation

10. Are there backflow preventing devices

○ Yes
○ No

11. The water delivery systems are:

○ Disinfected
○ Maintained
○ Cleaned
○ Not maintained

12. When is the last irrigation

○ Same day as harvest
○ 1 day before harvest
○ 2–4 days before harvest
○ 5–7 days before harvest
○ More than 7 days before harvest
○ Depending on the weather
